# Education Level and Cigarette Smoking: Diminished Returns of Lesbian, Gay and Bisexual Individuals

**DOI:** 10.3390/bs9100103

**Published:** 2019-09-24

**Authors:** Shervin Assari, Mohsen Bazargan

**Affiliations:** 1 Department of Family Medicine, Charles R Drew University of Medicine and Science, Los Angeles, CA 90059, USA; 2 Department of Family Medicine, University of California Los Angeles, Los Angeles, CA 90095, USA; Mohsenbazargan@cdrewu.edu

**Keywords:** lesbian, gay, and bisexual (LGB), sexual orientation, minorities, sexual minorities, socioeconomic position, socioeconomic status, poverty status, education, smoking, tobacco use

## Abstract

*Background:* Education level is one of the strongest protective factors against high-risk behaviors such as cigarette smoking. Minorities’ Diminished Returns (MDRs), however, suggest that the protective effects of education level tend to be weaker for racial and ethnic minority groups relative to non-Hispanic White people. Only two previous studies have shown that MDRs may also apply to lesbian, gay, and bisexual (LGB) individuals; however, these studies have focused on outcomes other than tobacco use. *Aims:* To compare LGB and non-LGB American adults for the effects of education level on cigarette-smoking status. *Methods:* Population Assessment of Tobacco and Health (PATH; 2013) entered 31,480 American adults who were either non-LGB (*n* = 29,303, 93.1%) or LGB (*n* = 2,177; 6.9%). The independent variable was education level. The dependent variable was current established cigarette smoking. Race, ethnicity, age, gender, poverty status, employment, and region were the covariates. LGB status was the moderator. *Results:* Overall, individuals with higher education level (odds ratio (OR) = 0.69) had lower odds of current established smoking. We found a significant interaction between LGB status and education level suggesting that the protective effect of education level on smoking status is systemically smaller for LGB people than non-LGB individuals (OR for interaction = 1.19). *Conclusions:* Similar to the patterns that are shown for racial and ethnic minorities, MDRs can be observed for the effects of education level among sexual minorities. In the United States, highly educated LGB adults remain at high risk of smoking cigarettes, a risk which is disproportionate to their education level. In other terms, high education level better helps non-LGB than LGB individuals to avoid cigarette smoking. The result is a relatively high burden of tobacco use in highly educated LGB individuals.

## 1. Background

Despite showing a downward trend in the US in recent years, cigarette smoking is still the leading cause of death and premature mortality in the United States [1,2,3]. Tobacco is responsible for the deaths of 480,000 Americans each year [4]. In addition, about 16 million Americans suffer tobacco-related chronic diseases [4]. Such morbidity and mortality costs the US society more than 300 billion dollars each year in direct and indirect costs [5]. 

Tobacco burden, however, has a larger hit on socially marginalized groups [6,7,8,9,10]. With an uneven decline in tobacco burden over the past few years, tobacco use has shifted from a mainstream to a concentrated public health problem [11]. Tobacco is now a health challenge for the marginalized people defined by sexual orientation, race, ethnicity, and socioeconomic status (SES) [11]. A higher burden of tobacco use in marginalized groups should be regarded as a major threat to the progress that US has already made in controlling the tobacco burden [11].

In the US, sexual minorities [12,13,14], racial and ethnic minority groups [6,7,8,9,10], and low SES individuals [15,16,17] suffer a disproportionately higher burden of tobacco use. Some evidence suggests that some of the smoking gap between the marginalized and non-marginalized populations (e.g. SES) is increasing [11] [17,18,19]. As a result of being a target of predatory tobacco marketing strategies [20,21,22], marginalized populations are commonly exposed to environmental tobacco risk factors such as point-of-sale advertisement, retail display, coupons, and discounts [23]. As a result of increased exposure to risk factors [24] and low interest toward tobacco cessation programs [8,25,26], some of the above marginalized groups experience additional vulnerability to tobacco use. Sexual minority individuals are among populations that are specifically targeted by the tobacco industry [27,28,29]. 

According to the Minorities’ Diminished Returns (MDRs) theory [30,31] which is supported by an extensive body of empirical research [32,33,34], at least some of the tobacco-use disparities are due to “*less than expected*” protective effects of education level on tobacco use in the members of the marginalized groups [32,33,34]. This model proposes that: (a) social inequalities in tobacco burden are not all due to SES disparities across social groups, but at least some are due to the MDRs (i.e. smaller health effects of SES indicators for the member of the majority groups, compared to the general population); and (b) the tobacco-use inequalities between the marginalized and non-marginalized groups widen, instead of narrowing, at higher education levels. As a result, the solution to tobacco disparities should go beyond reducing the inequalities in SES by addressing the societal ad structural factors that increase the tobacco use of highly educated members of social groups with a stigmatizing identity [32,33,34]. 

Although MDRs are documented for tobacco use, meaning that the members of the minority groups remain at higher risk of tobacco use despite their high education level [32,33,34], most of this research has defined minority status based on race and ethnicity [32,33,34]. We are only aware of one study that has shown MDRs for sexual minority populations , however that study has focused on obesity not smoking as an outcome [35]. In that study, lesbian, gay, and bisexual (LGB) individuals remained at high risk of obesity despite their high education level. For non-LGB individuals, however, education level reduced risk of obesity [35]. Authors argued that minority status, broadly defined, is associated with marginalization and stigmatization, and these processes may reduce the health gain that is expected to follow education level [35]. More research, however, is needed on MDRs of education level of tobacco use of LGB individuals. 

This study was performed to compare LGB and non-LGB individuals for the association between education level and tobacco use in a nationally representative sample of American adults. In line with the previous literature on MDRs [30,31], we expected smaller protective effect of education level on tobacco use for LGB compared to non-LGB individuals. As a result of such MDRs, we expected high tobacco use among highly educated LGB people. 

## 2. Methods

### 2.1. Design and Settings

This is a secondary analysis of the Population Assessment of Tobacco and Health (PATH) data. Funded by the U.S. National Institutes of Health (NIH) and the Food and Drug Administration (FDA), PATH is the state-of-the art study on tobacco use of Americans. PATH has enrolled about 49,000 people 12 years or older, who may or may not use tobacco at baseline. Wave 1 data were collected in 2013–2014. Although PATH also has youth data, this study only includes adults. We used publicly available PATH data sets from the Inter-university Consortium for Political and Social Research (ICPSR).

### 2.2. Sample and Sampling

The inclusion criteria in the PATH-Adult study was (1) civilian, (2) non-institutionalized, (3) U.S. population, and (4) 18 years of age and older. Sampling in the PATH study was a four-stage stratified area probability sample. The first stage was selection of a stratified sample (n = 156) of geographical primary sampling units (PSUs). These PSUs were either a county or a group of counties. The second stage sampled smaller geographical segments in each PSU. The third-stage sampled residential addresses, using the U.S. Postal Service Computerized Delivery Sequence Files. The fourth stage was the selection of one person from each sampled household. 

### 2.3. Analytical Sample

The current analysis is limited to the adults who had valid data on sexual orientation and tobacco use. Our final analytical sample was 31,480 individuals.

### 2.4. Study Variables

All study variables were measured at an individual level. The independent variable was education level. The dependent variable was current established smoking status. Race, ethnicity, gender, age, poverty status, employment, and region were the covariates. Sexual orientation was the moderator.

*Covariates.* Demographic factors such as age, gender, race, ethnicity, and region were measured. Age was a continuous measure, ranging from 1 to 7: 1) “18 to 24 years old”, 2) “25 to 34 years old”, 3) “35 to 44 years old”, 4) “45 to 54 years old”, 5) “55 to 64 years old”, 6) “65 to 74 years old”, and 7) “75+ years old”. Gender was a dichotomous variable (Male = 1, Female = 0). Race was self-identified and operationalized as a dichotomous variable: (Blacks = 1, Whites = 0). Ethnicity was also self-identified and operationalized as a dichotomous variable: (Hispanic = 1, Non-Hispanic = 0). Poverty status was dichotomous variable above 100% federal poverty line = 1, below 100% federal poverty line = 0. 

*Independent Variable.* Education level was a six-level continuous variable as below: 1) Less than high school, 2) General Educational Development (GED), 3) High-school graduate, 4) Some college, 5) Bachelor’s degree, and 6) Advanced degree. This variable was treated as a continuous measure.

*Outcome.* Our outcome was established current smoking (i.e. smoked 100 cigarettes and smokes currently). 

*Moderator.* Sexual orientation was self-identified by asking the individuals to report their sexual orientation. This variable was operationalized as non-LGB = 0 and LGB = 1. One question was used to measure sexual orientation. Participants were then asked, “Do you think of yourself as: (1) “Lesbian or gay”, (2) “Straight, that is not lesbian or gay”, (3) “Bisexual”, or (4) “Something else”. If a participant selected “something else”, then they were probed for more information. Based on the above responses, participants were recoded to LGB and non-LGB individuals [36,37]. 

### 2.5. Statistical Analysis

We analyzed the data using SPSS 23.0 (IBM Corporation, Armonk, NY, USA). We applied Taylor series linearization to re-estimate the variance of our variables using survey design variables such as weight, PSU, cluster, and strata. As a result of applying weight, the results are generalizable to the U.S. general population. For data analysis, first, we examined the distribution of our categorical and continuous variables. For univariate analysis, we used frequency tables. For continuous measures, we reported means and standard deviations (SDs). We then ruled out collinearity between independent variables and confounders such as education level, race, ethnicity, and poverty status. For bivariate analysis, we used the Pearson correlation test in the pooled sample and also by LGB status. For multivariable analysis, we applied binary logistic regression. We ran two logistic regression models: *Model 1* only had the main effects. *Model 2* also included an interaction term between education level and LGB status. We also ran two group specific models: *Model 3* was performed in non-LGB people. *Model 4* was performed in LGB people. We reported odds ratio (OR), 95% confidence interval (CI), and p values.

### 2.6. Ethics

All the participants provided written informed consent. The Institutional Review Board of the Westat approved the PATH study protocol.

## 3. Results

### 3.1. Descriptive Statistics

This study included 31,480 American adults who were either non-LGB (*n* = 29303, 93.1%) or LGB (*n* = 2177; 6.9%). Table 1 shows descriptive statistics of the overall sample as well as by sexual orientation. 

### 3.2. Bivariate Analysis

Table 2 shows bivariate correlations in the overall (pooled) sample and also based on LGB status. In the pooled sample, LGB status was positively correlated with race (Black), ethnicity (Hispanic), education level, and current smoking. LGB status was negatively correlated with gender (male) and age. Current smoking staus was negatively correlated with educational level and living out of poverty status but not with full-time employment. Bivariate and inverse correlation between education level and current smoking was stronger in non-LGB (r = −0.21) than LGB (r = −0.09) individuals. 

### 3.3. Multivariable Models in the Pooled Sample

Table 3 presents the summary of the results of two logistic regression models with education level as the independent variable (IV) and current smoking as the dependent variable (DV). Both models were estimated in the overall sample. *Model 1* only entered the main effect of education level and covariates. *Model 2,* however, also added an interaction term between sexual minority status and education level. 

Based on *Model 1*, high education level was associated with lower odds of being a current smoker. *Model 2* showed a statistically significant interaction between LGB status and education level on current smoking, suggesting that high education level has smaller protective effect on current smoking for LGB than non-LGB individuals.

### 3.4. Multivariable Models in Lesbian, Gay, and Bisexual (LGB) and non-LGB individuals

Table 4 presents the results of two stratified logistic regression models with education level as the independent variable and current smoking as the dependent variable. *Model 3* and *Model 4* were estimated in non-LGB and LGB people, respectively. 

Based on *Model 3*, high education level was associated with lower odds of current established smoking in non-LGB individuals. *Model 4* also showed the same pattern for LGB individuals. The magnitude of the protection, however, was larger for non-LGB (OR = 0.69) than LGB (OR = 0.81) individuals.

## 4. Discussion 

High education level was associated with lower odds of current established smoking, however, LGB and non-LGB people differed in such protection. Our results suggest that high education levels may have a smaller protective effect against cigarette smoking for LGB than non-LGB individuals. 

Built on our previous work showing that high SES racial and ethnic minority individuals remain at an increased risk of substance use compared to high SES Whites [33,34,38,39], we showed a high risk of tobacco use in highly educated LGB individuals. These patterns are similar to what is shown for a wide range of associations between SES indicators and health outcomes [30,31], however, most of the previous work on MDRs is for Blacks and Hispanics. The effects of education [40], income [41], employment [42], and marital status [43] on obesity [44], depression [45], anxiety [43], self-rated health [40], and chronic disease [46] are all smaller for racial and ethnic minorities than Whites. In one study, education level better prevented against obesity in non-LGB than LGB people [35]. The current study is the first to document the MDRs of education level on tobacco use for the LGB community.

In a study of LGBs in Nebraska, 763 respondents reported their social determinants of health (SDOHs) as well as smoking status. The prevalence of current smoking was 26%. Some SDOHs were predictors of smoking status in this population. However, after controlling for known risk factors of smoking in a logistic regression model, the SDOH variables were not more related to smoking status [47]. Their finding can be attributed to the MDRs of SDOH, which is in line with our findings on the diminished returns of education, one of the strongest SDOHs. 

There is a need to understand the structural and behavioral mechanisms that explain additional risk of tobacco use in highly educated socially marginalized individuals. Potential mechanisms may include stigmatization that generates stress and discrimination [35]. Predatory marketing practices that particularly target members of the minority groups may be one of the mechanisms that cause disparities in tobacco use [27,28,29,48,49]. We argue that such predatory practices may specifically generate MDRs, meaning that predatory marketing and advertising may cause disproportionately higher risk of tobacco use among high SES minority individuals. If that is the case, introducing more restrictive marketing policies that ban point-of-sale advertisement and flavoring may not only reduce overall smoking rates but may reduce inequalities based on marginalization status. This hypothesis, however, needs more research [27].

Multiple studies inside [47] and outside the U.S. [50] have shown higher tobacco use in the LGB communities. High prevalence of tobacco use in LGBs is not limited to the U.S. and holds for several other countries. However, we still do not know whether LGB and non-LGB individuals outside the U.S. also differ in the returns of educational attainment. Currently, our findings are not yet transferable to other contexts such as Australia, Canada, and European countries. Most of the MDRs literature is limited to the U.S. context. There is a need for cross-national studies that can compare various settings for diminished returns of SES indicators across marginalized groups. The very same marginalizing identity may be differently penalized across settings. Such comparative data would determine if the MDRs are globally replicable or they are only relevant beyond the U.S. context. Such research will help deepen our understanding on the contribution of SDOHs as contributors to tobacco health disparities, globally and locally.

Diminished returns of educational attainment on smoking in LGBs might be due to high exposure or vulnerability of the LGB people to tobacco-related messages and advertisements [29]. We know that LGB people (particularly LGBT smokers) are more likely to be exposed to and interact with tobacco-related messages on social media than non-LGBT people [51]. In addition, LGB people may also have lower perceived harm associated with some tobacco products [52]. LGBs are less likely to have health insurance, which may be needed for tobacco treatment counseling [53]. Some evidence also suggests that LGB people may have higher vulnerability to tobacco promotion advertisements [54]. Although LGBs and non-LGBs are similarly exposed to advertisements for quit lines, they are less likely to be aware of availability of quit lines [53,55]. As a result, LGBs are five times less inclined to call quit lines for smoking [56]. Some of our strategies to prevent tobacco use may be less effective for LGB people [51]. All these studies suggest that the very same investment on prevention (if not tailored) may result in diminished returns for the LGB individuals [53,55].

### 4.1. Implications 

Elimination of tobacco-related disparities is a strategic priority for the FDA. “Understanding why people become susceptible to using tobacco products” is a priority for the FDA. The results of this study may suggest some additional insight to one of the mechanisms by which marginalized groups remain at high risk of tobacco use. Our results suggest that public policies that can reduce tobacco-related disparities are not just those which reduce SES gaps but also those that address MDRs of education level. Setting more restrictive policies that tighten tobacco regulations is needed. Research has shown that such regulations are very acceptable to the U.S. public [57]. Thus, more restrictive policies are not seen by the U.S. public as a threat to their freedom and agency [57]. We already know that policies and regulations are more effective than individual-level interventions that overemphasize individual choices [57]. 

There is a need for policies at a national and local levels that can reduce the existing inequalities and disparities in tobacco use between the marginalized and non-marginalized groups, and reducing MDRs of SES are among them [31,33,34,39,40,41,43,44,58,59]. There is need to study how discounts, coupons, and flavoring increase the tobacco use of highly educated individuals who are a member of minority groups [33,34]. It is also unknown how tobacco regulations and policies can be used to undo the MDRs of education level on tobacco use for highly educated individuals that are members of marginalized groups [33,34,38,39]. We still do not know how marketing strategies disproportionately impact the LGB communities. To undo social disparities in tobacco use, there may be a need to ban predatory marketing that target marginalized people.

### 4.2. Limitations 

This study had some methodological limitations. The cross-sectional design of our data does not allow causal inferences. Sample size was imbalanced in non-LGB and LGB groups. This issue, however, only impacts our stratified models not our pooled sample model with the interaction terms. Income, marital status, and area-level SES were missing. This study did not measure neighborhood characteristics, exposure to tobacco marketing, and density of tobacco retailers in the area. Despite these limitations, we believe this study still extends the existing tobacco disparities literature by education level and sexual minority status. 

Some LGB-related factors such as sexual orientation and identity are significant contributors to tobacco use in the LGB community [60]. However, we could not control for such variables simply because those variables are irrelevant to non-LGB people. Future within-group research may explore LGB-related factors that reduce diminished returns of education for LGB people. As gender, sex, and sexual identity alter tobacco use, exposure, and response to tobacco advertisements, future research should explore how MDRs vary based on these social constructs. Given these complexities, future analyses may be stratified by these variables [50]. Another limitation was that we did not include trans-gender people who are at very high risk of smoking. For example, around a fifth of female-to-male transgender Australians smoke daily [61]. Future research should also include transgender people.

## 5. Conclusion

In the United States, education level better helps non-LGB than LGB individuals to stay healthy and avoid high-risk behaviors such as cigarette smoking. The result is additional risk of tobacco use in highly educated LGB people. Policy makers should not reduce the problem of tobacco disparities to the inequalities in SES as some mechanisms result in inequalities between marginalized and non-marginalized people in high SES levels. 

## Figures and Tables

**Table 1:** Descriptive statistics.

	All		Non-LGB		LGB	
	**n**	**%**	**n**	**%**	**n**	**%**
LGB						
No	29,303	93.1	29,303	100.0	-	-
Yes	2177	6.9	-	-	2177	100.0
Race						
White	23,303	82.6	21,768	82.8	1535	80.8
Black	4892	17.4	4527	17.2	365	19.2
Ethnicity *						
Non-Hispanic	25,733	82.9	24,079	83.3	1654	77.3
Hispanic	5305	17.1	4820	16.7	485	22.7
Gender *						
Women	15,534	49.3	14,091	48.1	1443	66.3
Men	15,946	50.7	15,212	51.9	734	33.7
Region						
West	4907	15.6	4553	15.5	354	16.3
Northeast	7527	23.9	7062	24.1	465	21.4
Midwest	11,919	37.9	11,113	37.9	806	37.0
South	7127	22.6	6575	22.4	552	25.4
Poverty status *						
Living in poverty	9739	33.9	8776	32.8	963	47.6
Living out of poverty	19,001	66.1	17942	67.2	1059	52.4
Full-time employment *						
No	17,286	54.9	15,965	54.5	1321	60.7
Yes	14,194	45.1	13,338	45.5	856	39.3
Current smoker *						
Non-smoker	20,292	64.5	19,054	65.0	1238	56.9
Smoker	11,188	35.5	10,249	35.0	939	43.1
	Mean	SD	Mean	SD	Mean	SD
Age (1–7) *	2.93	1.74	2.98	1.75	2.29	1.48
Education (1–6) *	3.53	1.37	3.54	1.37	3.39	1.40

* *p* < 0.05 for comparison of LGB and non-LGB; LGB: Lesbian, Gay, and Bisexual.

**Table 2:** Bivariate correlations.

	1	2	3	4	5	6	7	8	9
**All**									
1 Sexual orientation (LGB)	1	0.01 *	0.04 **	−0.09 **	−.10 **	−0.08 **	−0.03 **	−0.03 **	0.04 **
2 Race (Black)		1	−0.11 **	−0.02 **	−.03 **	−0.17 **	−0.05 **	−0.10 **	−0.04 **
3 Ethnicity (Hispanic)			1	0.00	−0.14 **	−0.17 **	−0.01	−0.18 **	−0.08 **
4 Gender (Male)				1	−0.00	0.07 **	0.16 **	−0.05 **	0.03 **
5 Age (Years)					1	0.21 **	−0.08 **	0.03 **	0.02 **
6 Living out of poverty						1	0.28 **	0.36 **	−0.11 **
7 Full-time employment							1	0.19 **	−0.01
8 Education level (1-6)								1	−0.20 **
9 Current established smoking (Yes)									1
**Non-LGB**									
1 Sexual orientation (LGB)	-	-	-	-	-	-	-	-	
2 Race (Black)		1	−0.11 **	−0.01 *	−0.03 **	−0.17 **	−0.05 **	−0.10 **	−0.04 **
3 Ethnicity (Hispanic)			1	.00	−0.14 **	−0.17 **	−0.01 *	−0.18 **	−0.08 **
4 Gender (Male)				1	−0.02 **	0.06 **	0.16 **	−0.06 **	0.04 **
5 Age (Years)					1	0.21 **	−0.09 **	0.02 **	0.02 **
6 Living out of poverty						1	0.28 **	0.36 **	−0.11 **
7 Full-time employment							1	0.19 **	−0.01
8 Education level (1-6)								1	−0.21 **
9 Current established smoking (Yes)									1
**LGB**									
1 Sexual orientation (LGB)	-	-	-	-	-	-	-	-	
2 Race (Black)		1	−0.12 **	−0.08 **	−0.07 **	−0.16 **	−0.07 **	−0.12 **	−0.06 *
3 Ethnicity (Hispanic)			1	0.04 *	−0.06 **	−0.17 **	0.02	−0.23 **	−0.11 **
4 Gender (Male)				1	0.15 **	0.14 **	0.13 **	0.08 **	−0.05 *
5 Age (Years)					1	0.23 **	0.07 **	0.10 **	0.04 *
6 Living out of poverty						1	0.30 **	0.40 **	−0.06 **
7 Fulltime employment							1	0.21 **	−0.01
8 Education level (1-6)								1	−0.09 **
9 Current established smoking (Yes)									1

LGB: Lesbian, Gay, and Bisexual; * *p* < 0.05 ** *p* < 0.01 (Pearson Correlation Test).

**Table 3:** Logistic regressions on current smoking models in the pooled sample.

	OR	95% CI	*p*
**Model 1 (All)**			
Sexual orientation (LGB)	1.49	1.34–1.65	< 0.001
Race (Black)	0.59	0.54–0.63	< 0.001
Ethnicity (Hispanic)	0.41	0.38–0.45	< 0.001
Gender (Male)	1.06	1.00–1.12	0.034
Age (Years)	1.03	1.01–1.04	0.002
Region			< 0.001
West	1.00	-	-
Northeast	1.14	1.05–1.25	0.002
Midwest	1.04	0.96–1.13	0.330
South	0.81	0.74–0.89	< 0.001
Living out of poverty	0.68	0.64–0.73	< 0.001
Full-time employment	1.23	1.16–1.31	< 0.001
Education level (1–6)	0.69	0.68–0.71	< 0.001
Constant	2.55		< 0.001
**Model 2 (All)**			
Sexual orientation (LGB)	0.82	0.62–1.07	0.149
Race (Black)	0.59	0.54–0.63	< 0.001
Ethnicity (Hispanic)	0.42	0.38–0.45	< 0.001
Gender (Male)	1.06	1.00–1.12	0.046
Age (Years)	1.03	1.01–1.04	0.002
Region			< 0.001
West	1	-	-
Northeast	1.14	1.05–1.25	0.002
Midwest	1.04	0.96–1.13	0.339
South	0.81	0.74–0.89	< 0.001
Living out of poverty	0.68	0.64–0.73	< 0.001
Full-time employment	1.23	1.16–1.31	< 0.001
Education level (1–6)	0.69	0.67–0.70	< 0.001
LGB × Education level	1.19	1.11–1.28	< 0.001
Constant	2.68		< 0.001

SE: Standard Error; CI: Confidence Interval; OR: Odds Ratio; LGB: Lesbian, Gay, and Bisexual.

**Table 4:** Logistic regression models on current smoking by sexual orientation.

	OR	95% CI	*P*
**Model 3 (Non-LGB)**			
Race (Black)	0.59	0.54–0.64	< 0.001
Ethnicity (Hispanic)	0.42	0.38–0.46	< 0.001
Gender (Male)	1.07	1.01–1.13	0.019
Age (Years)	1.02	1.01–1.04	0.008
Region			< 0.001
West	1.00	-	-
Northeast	1.16	1.06–1.27	0.001
Midwest	1.04	0.96–1.14	0.310
South	0.81	0.74–0.89	< 0.001
Living out of poverty	0.68	0.63–0.73	< 0.001
Full-time employment	1.24	1.17–1.32	< 0.001
Education level (1–6)	0.69	0.67–0.70	< 0.001
Constant	2.66		< 0.001
**Model 4 (LGB)**			
Race (Black)	0.56	0.43–0.73	< 0.001
Ethnicity (Hispanic)	0.42	0.32–0.55	< 0.001
Gender (Male)	0.85	0.69–1.05	0.134
Age (Years)	1.09	1.01–1.16	0.019
Region			0.638
West	1	-	-
Northeast	0.98	0.71–1.36	0.907
Midwest	0.98	0.73–1.31	0.896
South	0.84	0.61–1.16	0.298
Living out of poverty	0.75	0.60–0.94	0.014
Full-time employment	1.11	0.90–1.37	0.337
Education level (1–6)	0.81	0.75–0.88	< 0.001
Constant	2.23		< 0.001

SE: Standard Error; CI: Confidence Interval; OR: Odds Ratio; LGB: Lesbian, Gay, and Bisexual.

## References

[1] McCarthy M. (2014). Smoking remains leading cause of premature death in US. BMJ.

[2] Samet J.M. (2013). Tobacco smoking: The leading cause of preventable disease worldwide. Thorac. Surg. Clin..

[3] Novick L.F. (2000). Smoking is the leading preventable cause of death and disability in the United States. J. Public Health Manag. Pract..

[4] CDC Smoking & Tobacco Use. Fast Facts.

[5] CDC Economic Trends in Tobacco. https://www.cdc.gov/tobacco/data_statistics/fact_sheets/economics/econ_facts/index.htm.

[6] Ellickson P.L., Orlando M., Tucker J.S., Klein D.J. (2004). From adolescence to young adulthood: Racial/ethnic disparities in smoking. Am. J. Public Health.

[7] Kayani N., Homan S.G., Yun S. (2010). Prevention. Racial disparities in smoking-attributable mortality and years of potential life lost-Missouri, 2003–2007. Morb. Mortal. Wkly. Rep..

[8] Trinidad D.R., Perez-Stable E.J., White M.M., Emery S.L., Messer K. (2011). A nationwide analysis of US racial/ethnic disparities in smoking behaviors, smoking cessation, and cessation-related factors. Am. J. Public Health.

[9] Soulakova J.N., Huang H., Crockett L.J. (2015). Racial/ethnic disparities in consistent reporting of smoking-related behaviors. J. Addict. Behav. Ther. Rehabil..

[10] Blumenthal D.S. (2017). Racial and ethnic disparities in smoking prevalence in Israel and the United States: Progress to date and prospects for the future. Isr. J. Health Policy Res..

[11] Drope J., Liber A.C., Cahn Z., Stoklosa M., Kennedy R., Douglas C.E., Henson R., Drope J. (2018). Who’s still smoking? Disparities in adult cigarette smoking prevalence in the United States. CA Cancer J. Clin..

[12] Hinds J.T., Loukas A., Perry C.L. (2019). Explaining sexual minority young adult cigarette smoking disparities. Psychol. Addict. Behav..

[13] Fish J.N., Turner B., Phillips G., Russell S.T. (2019). Cigarette Smoking Disparities Between Sexual Minority and Heterosexual Youth. Pediatrics.

[14] Hoffman L., Delahanty J., Johnson S.E., Zhao X. (2018). Sexual and gender minority cigarette smoking disparities: An analysis of 2016 Behavioral Risk Factor Surveillance System data. Prev. Med..

[15] Laveist T.A., Thorpe R.J., Mance G.A., Jackson J. (2007). Overcoming confounding of race with socio-economic status and segregation to explore race disparities in smoking. Addiction.

[16] Reid J.L., Hammond D., Driezen P. (2010). Socio-economic status and smoking in Canada, 1999–2006: Has there been any progress on disparities in tobacco use?. Can. J. Public Health.

[17] Zhang X., Martinez-Donate A.P., Jones N.R. (2013). Educational disparities in home smoking bans among households with underage children in the United States: Can tobacco control policies help to narrow the gap?. Nicotine Tob. Res..

[18] Reimer R.A., Gerrard M., Gibbons F.X. (2010). Racial disparities in smoking knowledge among current smokers: Data from the health information national trends surveys. Psychol. Health.

[19] Rock V.J., Davis S.P., Thorne S.L., Asman K.J., Caraballo R.S. (2010). Menthol cigarette use among racial and ethnic groups in the United States, 2004–2008. Nicotine Tob. Res..

[20] Terry-McElrath Y.M., Wakefield M.A., Emery S., Saffer H., Szczypka G., O’Malley P.M., Johnston L.D., Chaloupka F.J., Flay B.R. (2007). State anti-tobacco advertising and smoking outcomes by gender and race/ethnicity. Ethn. Health.

[21] Keeler C., Max W., Yerger V., Yao T., Ong M.K., Sung H.Y. (2017). The Association of Menthol Cigarette Use With Quit Attempts, Successful Cessation, and Intention to Quit Across Racial/Ethnic Groups in the United States. Nicotine Tob. Res..

[22] Giovenco D.P., Spillane T.E., Merizier J.M. (2018). Neighborhood differences in alternative tobacco product availability and advertising in New York City: Implications for health disparities. Nicotine Tob. Res..

[23] Anderson S.J. (2011). Marketing of menthol cigarettes and consumer perceptions: A review of tobacco industry documents. Tob. Control.

[24] Greaves L., Hemsing N. (2009). Women and tobacco control policies: Social-structural and psychosocial contributions to vulnerability to tobacco use and exposure. Drug Alcohol Depend..

[25] Cokkinides V.E., Halpern M.T., Barbeau E.M., Ward E., Thun M.J. (2008). Racial and ethnic disparities in smoking-cessation interventions: Analysis of the 2005 National Health Interview Survey. Am. J. Prev. Med..

[26] Tran S.T., Rosenberg K.D., Carlson N.E. (2010). Racial/ethnic disparities in the receipt of smoking cessation interventions during prenatal care. Matern. Child Health J.

[27] Soneji S., Knutzen K.E., Tan A.S.L., Moran M.B., Yang J., Sargent J., Choi K. (2019). Online tobacco marketing among US adolescent sexual, gender, racial, and ethnic minorities. Addict. Behav..

[28] Smith E.A., Thomson K., Offen N., Malone R.E. (2008). “If you know you exist, it’s just marketing poison”: Meanings of tobacco industry targeting in the lesbian, gay, bisexual, and transgender community. Am. J. Public Health.

[29] Dilley J.A., Spigner C., Boysun M.J., Dent C.W., Pizacani B.A. (2008). Does tobacco industry marketing excessively impact lesbian, gay and bisexual communities?. Tob. Control.

[30] Assari S. (2018). Health Disparities due to Diminished Return among Black Americans: Public Policy Solutions. Soc. Issues Policy Rev..

[31] Assari S. (2017). Unequal gain of equal resources across racial groups. Int. J. Health Policy Manag..

[32] Shervin A., Ritesh M. (2019). Diminished Return of Employment on Ever Smoking Among Hispanic Whites in Los Angeles. Health Equity.

[33] Assari S., Farokhnia M., Mistry R. (2019). Education Attainment and Alcohol Binge Drinking: Diminished Returns of Hispanics in Los Angeles. Behav. Sci..

[34] Assari S., Mistry R. (2018). Educational Attainment and Smoking Status in a National Sample of American Adults; Evidence for the Blacks’ Diminished Return. Int. J. Environ. Res. Public Health.

[35] Assari S. (2019). Education Attainment and ObesityDifferential Returns Based on Sexual Orientation. Behav. Sci..

[36] Johnson S.E., O’Brien E.K., Coleman B., Tessman G.K., Hoffman L., Delahanty J. (2019). Sexual and Gender Minority U.S. Youth Tobacco Use: Population Assessment of Tobacco and Health (PATH) Study Wave 3, 2015–2016. Am. J. Prev. Med..

[37] Wheldon C.W., Kaufman A.R., Kasza K.A., Moser R.P. (2018). Tobacco Use Among Adults by Sexual Orientation: Findings from the Population Assessment of Tobacco and Health Study. LGBT Health.

[38] Assari S., Lankarani M.M. (2016). Education and alcohol consumption among older Americans; black-white differences. Front. Public Health.

[39] Assari S., Mistry R. (2018). Erratum: Assari, S.; Mistry, R. Educational Attainment and Smoking Status in a National Sample of American Adults; Evidence for the Blacks’ Diminished Return. Int. J. Environ. Res. Public Health.

[40] Assari S. (2018). Blacks’ diminished return of education attainment on subjective health; mediating effect of income. Brain Sci..

[41] Assari S., Hani N. (2018). Household income and children’s unmet dental care need; Blacks’ diminished return. Dent J..

[42] Assari S. (2017). Whites but not blacks gain life expectancy from social contacts. Behav. Sci..

[43] Assari S., Caldwell C.H., Zimmerman M.A. (2018). Family Structure and Subsequent Anxiety Symptoms; Minorities’ Diminished Return. Brain Sci..

[44] Assari S., Thomas A., Caldwell C.H., Mincy R.B. (2018). Blacks’ diminished health return of family structure and socioeconomic status; 15 years of follow-up of a national urban sample of youth. J. Urban Health.

[45] Assari S., Caldwell C.H. (2018). High risk of depression in high-income African American Boys. J. Racial Ethn. Health Disparities.

[46] Assari S., Caldwell C.H. (2019). Family Income at Birth and Risk of Attention Deficit Hyperactivity Disorder at Age 15: Racial Differences. Children.

[47] Pelster A.D., Fisher C.M., Irwin J.A., Coleman J.D., McCarthy M.A. (2015). Tobacco Use and Its Relationship to Social Determinants of Health in LGBT Populations of a Midwestern State. LGBT Health.

[48] Lee J.G., Griffin G.K., Melvin C.L. (2009). Tobacco use among sexual minorities in the USA, 1987 to May 2007: A systematic review. Tob. Control..

[49] Stevens P., Carlson L.M., Hinman J.M. (2004). An analysis of tobacco industry marketing to lesbian, gay, bisexual, and transgender (LGBT) populations: Strategies for mainstream tobacco control and prevention. Health Promot. Pract..

[50] Shahab L., Brown J., Hagger-Johnson G., Michie S., Semlyen J., West R., Meads C. (2017). Sexual orientation identity and tobacco and hazardous alcohol use: Findings from a cross-sectional English population survey. BMJ Open.

[51] Emory K., Buchting F.O., Trinidad D.R., Vera L., Emery S.L. (2019). Lesbian, Gay, Bisexual, and Transgender (LGBT) View it Differently Than Non-LGBT: Exposure to Tobacco-related Couponing, E-cigarette Advertisements, and Anti-tobacco Messages on Social and Traditional Media. Nicotine Tob. Res..

[52] Nayak P., Salazar L.F., Kota K.K., Pechacek T.F. (2017). Prevalence of use and perceptions of risk of novel and other alternative tobacco products among sexual minority adults: Results from an online national survey, 2014–2015. Prev. Med..

[53] Ranji U., Beamesderfer A., Kates J., Salganicoff A., Henry J. (2014). Health and Access to Care and Coverage for Lesbian, Gay, Bisexual, and Transgender Individuals in the US.

[54] Navarro M.A., Hoffman L., Crankshaw E.C., Guillory J., Jacobs S. (2019). LGBT Identity and Its Influence on Perceived Effectiveness of Advertisements from a LGBT Tobacco Public Education Campaign. J. Health Commun..

[55] Fallin A., Lee Y.O., Bennett K., Goodin A. (2015). Smoking Cessation Awareness and Utilization Among Lesbian, Gay, Bisexual, and Transgender Adults: An Analysis of the 2009–2010 National Adult Tobacco Survey. Nicotine Tob. Res..

[56] Burns E.K., Deaton E.A., Levinson A.H. (2011). Rates and reasons: Disparities in low intentions to use a state smoking cessation quitline. Am. J. Health Promot..

[57] Feliu A., Filippidis F.T., Joossens L., Fong G.T., Vardavas C.I., Baena A., Castellano Y., Martinez C., Fernandez E. (2019). Impact of tobacco control policies on smoking prevalence and quit ratios in 27 European Union countries from 2006 to 2014. Tob. Control.

[58] Assari S., Caldwell C.H., Mincy R. (2018). Family Socioeconomic Status at Birth and Youth Impulsivity at Age 15; Blacks’ Diminished Return. Children.

[59] Assari S. (2018). Socioeconomic status and self-rated oral health; diminished return among hispanic whites. Dent. J.

[60] Delahanty J., Ganz O., Hoffman L., Guillory J., Crankshaw E., Farrelly M. (2019). Tobacco use among lesbian, gay, bisexual and transgender young adults varies by sexual and gender identity. Drug Alcohol Depend..

[61] Jones T., De Bolger A.d.P., Dune T., Lykins A., Hawkes G. (2015). Female-to-Male (FTM) Transgender People’s Experiences in Australia: A National Study.

